# Cervical cancer in the Newly Independent States of the former Soviet Union: Incidence will remain high without action

**DOI:** 10.1016/j.canep.2021.101944

**Published:** 2021-08

**Authors:** Ariana Znaor, Anton Ryzhov, Marilys Corbex, Marion Piñeros, Freddie Bray

**Affiliations:** a Cancer Surveillance Branch, International Agency for Research on Cancer, Lyon, France; b Taras Shevchenko National University of Kyiv, Ukraine; c World Health Organization Regional Office for Europe, Copenhagen, Denmark

Despite the preventability of cervical cancer, the global burden remains high with 604,000 cases and 342,000 deaths estimated in 2020 [1]. Incidence rates vary widely from 2 to 3 per 100,000 in certain countries in the Middle East to 50–80 per 100,000 in Eastern Africa [1,2]. In 2018, the WHO launched a Global Strategy for Cervical Cancer Elimination, with an aim to reduce the incidence rate of cervical cancer worldwide to 4/100,000 women-years [3]. The long-term prospects of preventing the disease have been shown in modelling studies, with girls-only HPV vaccination at 90 % coverage alongside twice-lifetime HPV-based screening potentially halving incidence in low- and middle-income countries (LMIC) by 2048 [4].

With the political momentum in place to eliminate the disease over the next decades via national scale-up of the triple (vaccination, screening and treatment) intervention strategy, we would like to highlight the current situation in the Newly Independent States (NIS) of the former Soviet Union.

Opportunistic annual cytology screening of broad age groups – frequently using Romanowsky-Giemsa staining – remains a common practice in many NIS [5]; while national estimates of cervical cancer in Kazakhstan, Kyrgyzstan, Republic of Moldova, the Russian Federation, Turkmenistan and Ukraine are at present about four times the threshold incidence rate set by the Cervical Cancer Elimination Strategy, ranging between 14 and 17/100,000 [1].

Using the national and subnational cancer registry data from four NIS for the period 2008–2017, we examined the time trends in recorded age-standardised (world) incidence rates of invasive cervical cancer. Rates are uniformly high, and trends appear either stable or slightly increasing over the 10-year period (Fig. 1). Given this profile and a slow pace of transition towards evidence-based practices across the region, we believe that these results are broadly representative of other NIS for which there is a paucity of surveillance data available. The Figure thus reveals the dismal public health perspective of this high incidence region at the present time.

**Fig. 1:**
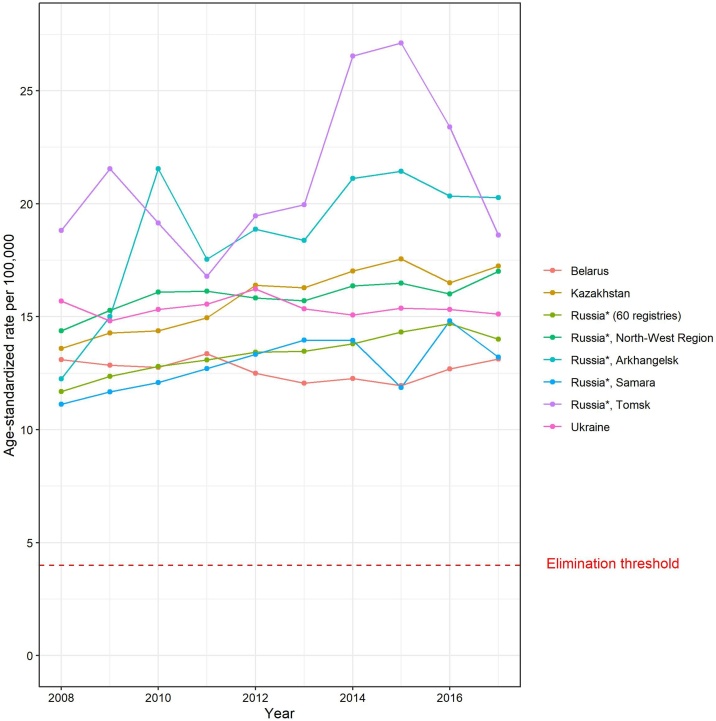
Trends in age-standardised incidence rates of invasive cervical cancer in selected population-based cancer registries in the NIS, 2008-2017. ASR –age-standardised rates (World Standard Population); Cervical cancer elimination threshold (4/100,000) is marked by a dashed red line. *Registries in the Russian Federation: 60 registries – Federal Registry data with 60 regions combined; North-West Region – North-Western Federal Region; Arkhangelsk – Arkhangelsk Region, Samara – Samara Region; Tomsk – Tomsk Region.

As a way forward towards cervical cancer elimination in the region, we advocate a radical shift in national policies in the NIS, away from opportunistic screening towards population-based, quality-assured HPV vaccination and HPV-based screening programmes. IARC partners with WHO Regional Office for Europe to improve screening policies and practices in the NIS, and to build capacity for quality assured screening and for population-based cancer registries, thus enabling the benchmarking of national progress in HPV-related cancer control in the forthcoming decades.

## Disclaimer

Where authors are identified as personnel of the International Agency for Research on Cancer / World Health Organization, the authors alone are responsible for the views expressed in this article and they do not necessarily represent the decisions, policy or views of the International Agency for Research on Cancer / World Health Organization.

## Declaration of Competing Interest

The authors report no declarations of interest.
